# Follow-up brain MRI of infants with and without congenital Zika virus infection: paving the way for a thorough understanding of the consequences of Zika virus infection

**DOI:** 10.1590/0100-3984.2024.57.e12

**Published:** 2024-12-11

**Authors:** Patricia Piazza Rafful, Sara Reis Teixeira

**Affiliations:** 1 MD, PhD, Department of Radiology, Children’s Hospital of Philadelphia, Philadelphia, PA, USA. Email: raffulp@chop.edu; 2 MD, PhD, Department of Radiology, Division of Neuroradiology, Children’s Hospital of Philadelphia, Assistant Professor of Radiology, Perelman School of Medicine, University of Pennsylvania, Philadelphia, PA, USA. Email: teixeiras@chop.edu

When the World Health Organization declared the Zika virus infection outbreak as a public health emergency in early 2016, many of the cases in the Americas had already occurred, with devastating consequences, such as increased numbers of stillbirths and children born with microcephaly^(1,2)^. To date, 89 countries and territories have reported cases of mosquito-borne Zika virus infection^(3)^. Following the epidemic of such infection in the northeastern region of Brazil, other outbreaks surged through different regions of the country^(4,5)^. Although the initial outbreak happened almost a decade ago, it is important not to forget the lessons learned. The increasing vulnerability of the population might lead to other outbreaks in the future. Data from the Brazilian National Ministry of Health show an increase in the number of probable cases of Zika virus infection in the last year, most notably in the southeastern and central-west regions^(5)^. Therefore, continued surveillance and investigation of children affected by the Zika virus are still warranted. In addition, lessons learned from Zika virus research may pave the way to accelerate the response to investigate vertical transmission of other zoonoses, such as infection with the Oropouche virus^(6)^.

In a study recently published in Radiologia Brasileira, Santos et al.^(7)^ investigated the evolution of brain MRI findings in infants perinatally exposed to the Zika virus, with and without congenital Zika syndrome (CZS). The authors also aimed to correlate the imaging findings with clinical and neurological outcomes at one year after the initial brain MRI. The study population was composed of 36 infants who underwent serial neurological examinations up to 24 months of age. The median age at the initial brain MRI was 12 months, and the follow-up brain MRI was performed one year later. Twenty-five infants (69.4%) met the criteria for CZS. Of those 25 infants with CZS, 18 (72%) presented with classic brain MRI findings consistent with congenital Zika virus infection^(8)^ and seven (28%) had mild nonspecific MRI findings. All 18 of the infants with CZS and classic MRI findings had moderate-to-severe neurodevelopmental impairment. Among the 11 infants without CZS, brain MRI showed periventricular white matter signal abnormalities in five (45%), all with mild neurodevelopmental changes. The six remaining infants in that group (55%) had a normal brain MRI, all with normal neurological examinations. Follow-up imaging one year later did not show any statistically significant difference in comparison with the initial brain MRI. This study^(7)^ adds to the literature critical information about brain MRI findings in infants without CZS, an underexplored group. The authors showed that despite mild brain MRI abnormalities, such as nonspecific periventricular white matter hyperintensities, more than a third of the infants without CZS were at risk of impaired neurodevelopment. In addition, with the unique approach of evaluating the evolution of imaging changes with a follow-up brain MRI after one year, this study showed that there was no evolution of brain abnormalities except for interval myelination. One of the limitations of the study, as the authors mention, is that the sample included a small number of infants without CZS. This may preclude further investigation of whether the neurological abnormalities found in such infants are overestimated, given that some bias may play a role. What the study does not address is the long-term follow-up of children with and without CZS.

Since the Zika virus infection outbreak in 2015, research efforts have dedicated resources to investigate multiple aspects of the infection, from basic science with *in vitro* studies to epidemiological studies trying to find associations between the infection in pregnant women and their outcomes. *In vitro* studies demonstrated the predilection of the Zika virus for and damage to the neural progenitors^(9)^. The Zika virus has been described as a causative agent of microcephaly in previous case–control studies^(10)^. It is now well known that Zika virus infection in the early stages of pregnancy is associated with more severe fetal brain damage. In addition, robust data from an individual participant data meta-analysis^(11)^ showed that nearly one third (31.5%) of infants exposed to the Zika virus *in utero* develop any abnormalities in the first years of life, regardless of the presence of microcephaly. In that same meta-analysis^(11)^, the risk of any brain imaging abnormality among such infants was found to be 7.9%. However, nearly a decade after the outbreak of Zika virus infection in the Americas and worldwide, not all questions regarding the congenital Zika virus infection have been answered. What is the long-term neurodevelopment of children without microcephaly and with or without CZS? Will the minor neurological abnormalities found in infants without CZS at 24 months of age severely affect their long-term outcomes? Does early intervention help mitigate potential impairments? As radiologists, can we detect early brain imaging biomarkers that will help a multidisciplinary team improve care for these children? Ultimately, these questions remain unanswered. Researchers and established consortia among multiple research groups^(11)^ will help clarify some of these questions in the future.
